# Supplementary material for: All-at-once RNA folding with 3D motif prediction framed by evolutionary information
Source: Nat Methods. 2025 Oct 3;22(10):2094–106. doi: 10.1038/s41592-025-02833-w (PMC12510878; doi:10.1038/s41592-025-02833-w)
Supplement: Supplementary file 3 — Source code to run CaCoFold-R3D. Data and results. [file 41592_2025_2833_MOESM3_ESM.gz › supplemental_material/results/CaCoFold-R3D/CaCoFold-R3D_Rfam_vshuffle/RF02403_Afu_328.R2R.sto.pdf]

RF02403_Afu_328

5′ – Y U ● ● A U G A U G A G C A R U U U Y A U U A Y U A G U U A U C C C U G G C A G A A ● Y A ● ● C U G U U G A U A A C ● Y A ● ○ ● C U C U G A A ● ●
